# Predictors of Retinal Nerve Fiber Layer Parameters Following Scleral Buckling Surgery in Primary Rhegmatogenous Retinal Detachment

**DOI:** 10.7759/cureus.21754

**Published:** 2022-01-31

**Authors:** Punita K Sodhi, Alka Yadav, Ekta Shaw, Sushil Kumar, Nandini Sharma, Shantanu Sharma

**Affiliations:** 1 Ophthalmology, Guru Nanak Eye Centre and Maulana Azad Medical College, New Delhi, IND; 2 Community Medicine, Maulana Azad Medical College, New Delhi, IND

**Keywords:** scleral buckling, rhegmatogenous retinal detachment, retinal nerve fiber layer, retinal detachment, predictors, ganglion cell count

## Abstract

Objective: This study aimed to examine predictors of retinal nerve fiber layer (RNFL) parameters following scleral buckling (SB) surgery for primary rhegmatogenous retinal detachment (RRD) and to determine the influence of the magnitude of change in qualitative and quantitative parameters on RNFL.

Methods: In an observational prospective study, 40 subjects who underwent successful retinal reattachment with SB surgery done within one month of RRD were evaluated for the parameters of best-corrected visual acuity (BCVA), refractive error, intraocular pressure (IOP), axial length (AL), anterior chamber depth (ACD), angle opening distance (AOD 500 and AOD 750), trabecular iris surface area (TISA 500 and TISA 750), visual fields, and ganglion cell count (GCC) and RNFL before and three months after SB. We additionally noted qualitative factors like extent, location, and type of buckle; phakic status; and grade of proliferative vitreoretinopathy in the affected eye. The change in value of quantitative parameters was found. The influence of baseline values and magnitude of change of quantitative and qualitative parameters on average RNFL thickness and magnitude of change of RNFL thickness after SB was found.

Results: Post-SB, average RNFL thickness reduced from 108.58±20.38 microns to 103.73±17.98 microns (p =0.042). The baseline temporal upper (TU), temporal lower (TL), and nasal lower (NL) RNFL thickness (p=0.01, p=0.02, p=0.01, respectively) and total deviation (TD) values of visual fields (p=0.01) correlated positively while baseline GCC gross loss of volume (p=0.01) correlated negatively with post-operative RNFL thickness. The TU, TL, and NL RNFL thickness (p=0.04, p=0.01, p=0.01, respectively) and average GCC (p=0.04) correlated negatively with the magnitude of change in RNFL. The magnitude of change in baseline parameters after surgery was correlated with the magnitude of change in average RNFL thickness. It was noticed that change in AL (p<0.01), TISA 500 (p=0.02), TISA 750 (p<0.01), GCC focal loss of volume (p=0.02), and temporal RNFL thickness (p<0.01) correlated positively while the change in refractive error correlated negatively (p=0.04). Except for the grade of proliferative vitreoretinopathy (PVR) (p=0.04), none of the qualitative parameters, including extent, type, and location of the buckle; and phakic status, had a significant association with post-operative average RNFL thickness or magnitude of its change.

Conclusions: The predictors of average RNFL thickness following SB include AL; myopic shift; TISA; visual fields TD; average, TU, TL, and NL RNFL thickness; average GCC, gross and focal loss of volume; and grade of PVR. So an early surgery to prevent preoperative ganglion cell and RNFL loss and progression of PVR is recommended.

## Introduction

The retinal nerve fiber layer (RNFL) is the final pathway for carrying messages from the eye. Retinal nerve fiber layer thickness is directly related to visual functions including visual acuity (VA) [1,2], contrast sensitivity (CS) [1], color vision (CV) [2], and visual fields (VF) mean deviation (MD), and pattern standard deviation (PSD) [3]. The RNFL parameters measure the retinal axonal loss [1].

The scleral buckling (SB) has a vital role in the repair of rhegmatogenous retinal detachment (RRD) in young phakic patients, RD associated with dialysis, anterior retinal breaks, and also in conjunction with pars plana vitrectomy (PPV) in subjects having trauma or proliferative vitreoretinopathy (PVR). However, for promoting reattachment, it causes pressure effect in a circumferential manner onto the retinal layers with an encirclage band, buckle, and tight sutures [4]. The explants stretch the optic nerve [5], change the ocular blood flow [6], and interrupt choroidal venous drainage leading to ocular ischemia and peripheral field defect [7].

With modern diagnostic and surgical techniques, the success of detachment repair is >90%. Nevertheless, visual results do not reflect this anatomic success rate [8], and the rate of achievement of central VA of LogMAR +0.4 (20/50; 6/15) or better is only 42-60% [9]. To find the total gamut of RD effects on the inner retinal layers, we need to study the RNFL status both - following RD and after detachment surgery. It is well established that despite reattachment, changes in RNFL may cause low visual gains [5]. But there are few studies on RNFL in subjects undergoing SB surgery [5,10,11]. In this study, we conducted automated RNFL measurement before and after SB surgery using spectral-domain optical coherence tomography (SD-OCT). We examined various ocular parameters and determined the correlation/association of their values with average RNFL thickness and the extent of its change after SB surgery.

## Materials and methods

A prospective observational study, without control, was conducted at the Department of Ophthalmology, Guru Nanak Eye Centre and Maulana Azad Medical College, New Delhi, on subjects having primary RRD. The trial was registered after obtaining the Maulana Azad Medical College Institutional Ethics Committee clearance (identifier, CTRI/2020/02/023158; approval, F. No.17/IEC/MAMC/2018/). The subjects who had primary RRD of less than the duration of a month were willing to participate and had PVR up to grade C2 were enrolled consecutively for SB surgery. The subjects who had undergone previous ocular surgery (apart from uncomplicated cataract surgery) had significant media opacity like cataract and vitreous hemorrhage, other co-existing ocular diseases like glaucoma, uveitis, thyroid ophthalmopathy, optic atrophy, other retinal diseases, and systemic diseases like diabetes and central nervous system disease were excluded.

A brief history was obtained for the duration of symptoms, and an ocular examination was performed for baseline evaluation of visual functions. During dilated fundus examination, extent of RD, involvement of macula, location of tears/hole/dialysis, and PVR grade were noted down. In accordance with the Declaration of Helsinki, written informed consent was obtained from all patients.

The distance best-corrected visual acuity (BCVA) was recorded on early treatment diabetic retinopathy study (ETDRS) distance acuity charts under uniform illumination for each eye separately for each subject. The subjects underwent manual retinoscopy and auto-refractometer examination in dilated pupils, and following subjective refraction, the final prescription was given. The IOP was measured with the Goldman applanation tonometer (Appa applanation tonometer, Model AATM-5001; Chennai, India: Appasamy Associates). The axial length (AL) and anterior chamber depth (ACD) were measured on an optical biometer (Lenstar 900; Koeniz, Switzerland: Haag-Streit Lenstar) by experienced investigators who took four different measurements, each with a signal-to-noise ratio (SNR) of >2.0 per eye and final reading was taken from average of these. To find the change in the anatomy of anterior chamber angle due to the pressure of explants, parameters including angle opening distance at 500 microns from scleral spur (AOD 500), angle opening distance at 750 microns from scleral spur (AOD 750), trabecular iris surface area at 500 microns from scleral spur (TISA 500), and trabecular iris surface area at 750 microns from scleral spur (TISA 750) were measured on anterior segment optical coherence tomography (AS-OCT) equipment (RTVue; Model iVue 100 version 2.6; Freemont, CA: Optovue Inc.) [12]. The measurements were taken for nasal and temporal anterior chamber angles and the average of two readings was taken [13]. The visual fields (VF) were plotted using static automated white-on-white threshold perimetry (Swedish Interactive Threshold Algorithm {SITA} Standard 24-2, Humphrey Field Analyzer II; Jena, Germany: Carl Zeiss Meditec) to determine mean deviation (MD) values and pattern deviation (PSD) values. A VF was defined as reliable when fixation losses, false-positive, and false-negative errors were <10% [10].

We characterized the fovea in SD-OCT as characteristic foveal depression where there is a lack of retinal layers including nerve fiber layer, ganglion cell layer, inner nuclear layer, and inner plexiform layer [14]. The central foveal thickness (CFT) was measured as the distance between hyperreflective line corresponding to retinal pigment epithelium and internal limiting membrane [14]. The CFT was measured manually using manual calipers and verified through automated measurement on the OCT machine.

After pupil dilatation, the RNFL thickness was measured on posterior segment SD-OCT machine (RTVue; Model iVue 100 version 2.6). The same experienced doctor performed all the scans. To be included, images had to be centered at the optic disc for measuring peripapillary (i.e., optic nerve head) RNFL and centered at fovea to find RNFL thickness at 1.7 mm from center of the macula, with accurate segmentation, and with a signal strength of >15 dB [15,16]. The cup dimensions were measured during optic nerve head (ONH) scan by two separate observers and averaged to get the final reading. At macula, the average RNFL, superior RNFL, and inferior RNFL thickness and thickness in each of eight sectors namely superior nasal (SN), nasal upper (NU), nasal lower (NL), inferior nasal (IN), inferior temporal (IT), temporal lower (TL), temporal upper (TU), and superior temporal (ST) were found. The SB surgery was done in a conventional manner.

Surgical technique

The encirclage band (Style 240, 2.5 mm; Uxbridge, MA: Mira Inc.) and buckle were pre-soaked in antibiotic solution (gentamicin 40mg/ml) for 30 minutes in order to prevent infection. Following peritomy, two relaxing incisions were given in conjunctiva at 3 o’clock and 9 o’clock. Intermuscular septa were dissected and four bridle sutures on four recti were passed. The tear was indented with a cryoprobe and transscleral cryotherapy was done while watching reaction with an indirect ophthalmoscope. Local circumferential symmetrical buckle/tire (277/279) (Style 277, 7 mm and Style; 279, 9 mm; Uxbridge, MA: Mira Inc.) to the causative break was sutured to indent the retina at the region of tear and enough care was exercised to place tear on the height of buckle. Encirclage band 2.5 mm was passed through the groove of buckle and then below recti, and it passed through all quadrants at 13 to 15 mm from the limbus, with non-absorbable 5-0 Ethibond sutures (braided polyester, spatulated, one-fourth circle, 7.9 mm, Aurobond; Madurai, India: Aurolab) and two free ends were left in the quadrant opposite the tear/hole. The subretinal fluid (SRF) was not drained in shallow RRD to prevent retinal incarceration. When indicated (e.g., old RD, bullous RD), the SRF was drained using a 26-gauge hypodermic needle, and we applied cryotherapy to the site of drainage followed by support with buckle. The sterile air was injected into the vitreous cavity in order to accelerate SRF absorption by causing better apposition between the neuroepithelial layer and pigment epithelium. The central retinal artery was checked at end of surgery. The final tie to the encirclage band was given in the stipulated quadrant and opposition of band was done with a clove hitch knot. No vortex veins or muscles were damaged during the operation. The conjunctival peritomy was sutured in the area where relaxing incisions were given. Subconjunctival gentamycin and decadron were given. The pad and bandage were done for the eye following the instillation of ointment atropine. Intravenous mannitol was given to reduce the intraocular pressure.

All parameters were measured at the pre-operative stage. The posterior segment OCT and visual fields parameters, on account of the difficulty in assessment in the detached retina, were measured at one-week post-operative stage assuming their constancy within the one-week period (pre-operative stage to one-week post-operative stage) [10,14]. Only subjects with well-settled retina and having distance BCVA of at least logarithm of the minimum angle of resolution (LogMAR) +1.0 in the affected eye following scleral buckling surgery (SBS) were included.

Primary anatomical success was defined as the reattachment of the retina following SB surgery. Primary functional success was defined as VA maintained (BCVA improved or worsened by < 2 lines of Snellen chart) or VA increased (BCVA improved > 2 lines of Snellen chart) at three months of follow-up. The visual gain was found from the difference between pre- and post-operative LogMAR visual acuity. 

All study parameters, including CFT, were measured again three months post-surgery, and these values were compared with pre-operative values. The primary outcome parameter was RNFL parameters and their changes. The correlation and association for quantitative and qualitative parameters with average RNFL thickness and with magnitude of change in RNFL thickness were found.

Statistical analysis

The analysis was performed using IBM SPSS statistical software version 25.0 (Chicago, IL: IBM Corp.). Quantitative data were expressed in mean ± standard deviation for parametric data or median with interquartile range for non-parametric data. The comparison between pre-operative and post-operative RNFL thickness was made using paired t-test. A correlation was found between various ocular parameters and magnitude of their change, with post-operative RNFL thickness and magnitude of its change. Pearson’s test was used for parametric data and the Spearman correlation test was used for non-parametric data. For qualitative parameters (extent, type, and location of the scleral buckle; phakic/pseudophakic/aphakic status; and PVR grade), the difference between two comparable groups was tested by Mann-Whitney U test (Wilcoxon rank sum test) while for more than two groups Kruskal-Wallis H test was used followed by post hoc test. The p-value less than 0.05 at 95% confidence interval was taken as statistically significant (SS).

## Results

The present study is a prospective observational study without control in 40 eyes of 40 consecutive subjects for evaluating predictors of RNFL thickness and magnitude of its change following successful retinal reattachment with SB surgery in primary RRD.

The cohort comprised of 25 (62.5%) males and 15 (37.5%) females having a mean age of 42±15.8 years (range: 18-65 years). They presented with complaints of diminution of vision, flashes of light, and curtain falling in front of eyes. A total of eight out of 40 (20%) subjects had a history of trauma, six out of 40 (15%) had high myopia, while 26 out of 40 (65%) subjects had no definite cause related to RRD. The retinal detachment was “macula off” in all the subjects (100%). The mean duration of vision loss/RD before presentation in our subjects was 19.5±4.7 days.

The tears were located in the inferior (inferior, inferonasal, inferotemporal) quadrant in 16 subjects, superior (superior, superonasal, supero-temporal) quadrant in 21 subjects, and horizontal clock hours (temporal) in the remaining three subjects, and corresponding was the buckle position. There was one tear in 23 subjects, two in eight subjects, and multiple tears in nine subjects.

The mean RNFL average, RNFL superior, and RNFL inferior was 108.58±20.38 microns (range: 68-160 microns), 105.9±20.24 microns (range: 71-154 microns), and 109.78±23.96 microns (range: 68-196 microns) at pre-operative stage, and it reduced to 103.73±17.98 microns (range: 64-132 microns; p=0.042, SS), 95.55±16.85 microns (range: 64-129 microns; p=0.001, SS), and 103.18±25.19 microns (range: 11-147 microns; p=0.057, NSS) post-operatively. Thus, the magnitude of change was, respectively, 4.85±17.25 microns (4.47%), 10.35±17.03 microns (9.77%), and 6.6±25.89 microns (6.01%) in these quadrants. Table 1 shows the mean values and range for pre-operative and post-operative ocular parameters and the statistical significance of this mean difference.

**Table 1:** Mean change in values of ocular parameters following SB surgery. *RNFL parameters (in microns) were measured at one-week post-operative instead of pre-operative (as macula was “off” at pre-operative stage). **Statistically significant correlation. BCVA: best-corrected visual acuity; IOP: intraocular pressure; RNFL: retinal nerve fiber layer; NU: nasal upper quadrant; NL: nasal lower quadrant; IN: inferior nasal quadrant; IT: inferior temporal quadrant; TL: temporal lower quadrant; TU: temporal upper quadrant; ST: superior temporal quadrant; SB: scleral buckling

S. no.	Ocular parameters	Mean pre-operative value (range)	Mean post-operative value (range)	Mean difference	% Change	p-Value
1.	BCVA (LogMAR)	2.13±0.44 (1.3-2.8)	1.09±0.4 (0.4-2.3)	1.04±0.57	48.94	<0.001**
2.	IOP (mmHg)	11.48±2.29 (7-18)	14.43±3.00 (10-27)	2.95±1.72	25.71	<0.001**
3.	RNFL average thickness (microns)*	108.58±20.38 (68-160)	103.73±17.98 (64-132)	4.85±17.25	4.47	0.042**
4.	RNFL superior thickness (microns)*	105.9±20.24 (71-154)	95.55±16.85 (64-129)	10.35±17.03	9.77	<0.001**
5.	RNFL inferior thickness (microns)*	109.78±23.96 (68-196)	103.18±25.19 (11-147)	6.6±25.89	6.01	0.057
6.	NU thickness (microns)*	81.73±28.78 (15-158)	75.6±21.58 (39-128)	6.13±24.01	7.49	0.057
7.	NL thickness (microns)*	77.25±28.68 (32-148)	84.05±33.36 (32-194)	6.8±24.51	8.80	0.044**
8.	IN thickness (microns)*	130.23±32.41 (93-240)	128.75±44.19 (10-235)	1.48±42.84	1.13	0.414
9.	IT thickness (microns)*	146.8±46.14 (16-262)	136.25±45.14 (22-222)	10.55±51.13	7.19	0.100
10.	TL thickness (microns)*	87.93±32.06 (40-178)	94.55±36.9 (41-178)	6.63±47.23	7.53	0.190
11.	TU thickness (microns)*	92.6±29.82 (26-160)	86.73±25.3 (38-134)	5.88±26.99	6.34	0.088
12.	ST thickness (microns)*	124.13±31.42 (10-218)	113.18±26.45 (64-162)	10.95±36.22	8.82	0.032**
13.	OCT ONH RNFL average (microns)*	93.75±21.29 (60.56-131.82)	84.35±19.93 (50.12-122.67)	9.40±24.71	10.02	0.011**

There was an SS change in average, superior, NL and ST RNFL, and average OCT ONH RNFL. The RNFL grew thinner in all quadrants except NL and TL. However, there was a SS improvement in distance BCVA as log MAR values showed reduction and an increase in mean IOP after SB.

In addition to the above, the mean values of other pre-operative parameters like optic disc cup dimensions were 0.35±0.13, refractive error (RE) was minus 1.56±2.7 D (myopic), AL was 23.82±1.83 mm, ACD was 3.65±0.49 mm, AOD500 was 686.33±197.28 microns, AOD750 was 869.08±260.39 microns, TISA500 was 0.249±0.644 microns, and TISA750 was 0.428±0.125 microns. The mean value of VF MD was minus 12.21±6.43 and PSD was 4.47±3.92. The ganglion cell count (GCC) average was 109.96±20.53 microns, focal loss of volume (FLV) was 4.37±4.79, gross loss of volume (GLV) was 6.69±6.94, and ONH RNFL average was 88.31±18.38 microns.

Table 2 shows the correlation between pre-operative parameters with post-operative average RNFL thickness and the magnitude of its change following SB surgery. Except for pre-operative BCVA, IOP, and AL, all other parameters, including the magnitude of change in average RNFL thickness, were non-parametric (Table 2). We found a correlation of IT, TL, TU, and NL RNFL with average RNFL parameters as temporal RNFL (TRNFL) is an anatomical representative of the inner retinal layers of the fovea, and NL RNFL showed a significant change following surgery [15].

**Table 2:** Correlation between pre-operative ocular parameters with post-operative RNFL thickness and with change in RNFL thickness (post-operative minus pre-operative) in affected eye at three months after surgery. *Parametric parameters. **CMT was measured at post-operative three months. ***Statistically significant correlation. BCVA: best-corrected visual acuity; AOD: angle opening distance; TISA: trabecular iris surface area; MD: mean deviation; PSD: pattern standard deviation; OCT: optical coherence tomography; GCC: ganglion cell count; FLV: focal loss of volume; GLV: gross loss of volume; ONH: optic nerve head; RNFL: retinal nerve fiber layer; CFT: central foveal thickness; RD: retinal detachment

S. no.	Pre-operative quantitative parameters	Post-operative RNFL thickness (quantitative)		Magnitude of change in RNFL post-operative minus (-) pre-operative (non-parametric)	
		r-Value (correlation coefficient)	p-Value	r-Value (correlation coefficient)	p-Value
1.	Age of subject (years)	-0.29	0.07	0.18	0.24
2.	Duration of RD (days)	0.11	0.48	0.23	0.15
3.	Pre-operative BCVA (LogMAR)*	0.08	0.60	-0.18	0.26
4.	Intraocular pressure*	0.12	0.45	-0.01	0.96
5.	Cup dimensions	-0.05	0.73	0.04	0.79
6.	Magnitude of refractive error	0.01	0.97	0.23	0.14
7.	Axial length (mm)*	0.05	0.75	0.01	0.92
8.	Anterior chamber depth (mm)	-0.23	0.14	-0.01	0.90
9.	AOD 500 (microns)	0.10	0.52	-0.27	0.08
10.	AOD 750 (microns)	0.08	0.58	-0.17	0.27
11.	TISA 500 (microns)	0.06	0.67	-0.30	0.06
12.	TISA 750 (microns)	0.08	0.60	-0.27	0.09
13.	Visual field analysis (dB) MD	0.43	<0.01***	0.24	0.12
14.	Visual field analysis (dB) PSD	-0.26	0.09	-0.15	0.34
15.	OCT GCC average (microns)	0.18	0.25	-0.31	0.04***
16.	OCT GCC FLV	-0.19	0.22	-0.30	0.05***
18.	OCT GCC GLV	-0.37	0.01***	0.11	0.49
19.	OCT ONH RNFL average (microns)	0.27	0.08	-0.10	0.52
20.	OCT RNFL IT (microns)	0.24	0.12	-0.41	<0.01***
21.	OCT RNFL TL (microns)	0.37	0.01***	-0.50	<0.01***
21.	OCT RNFL TU (microns)	0.36	0.02***	-0.30	0.05***
22.	OCT RNFL NL (microns)	0.37	0.01	-0.50	0.01
Post-operative three months parameter					
23.	Post-operative CFT (microns)**	0.01	0.97	0.07	0.65

The pre-operative parameters including VF MD (p<0.01), GCC GLV (p=0.01), RNFL TL, TU and NL (p=0.01, p=0.02, p=0.01) had significant correlation with post-operative RNFL thickness. The GCC average (p=0.04), GCC FLV (p=0.05), RNFL IT, TL, TU and NL (p<0.01, p<0.01, p=0.05, p=0.01) correlated with magnitude of change in RNFL thickness in a significant manner. The ONH RNFL average did not have significant correlation with average RNFL (r=0.27; p=0.08) or with its change (r= -0.10; p=0.52). The CFT in the affected eye measured 264.55±66.32 microns (179-491 microns) at post-operative three months. It did not have a significant correlation with post-operative RNFL thickness (r=0.01; p=0.97) or with the extent of its change (r=0.07; p=0.65) (Table 2).

The post-operative mean values of optic disc cup dimensions increased to 0.35±0.15 (p=0.080), RE increased to -2.79±2.33 D (myopic shift; p<0.001), AL increased to 24.44±1.86 mm (p<0.001), ACD reduced to 3.49±0.49 mm (p<0.001), AOD500 reduced to 649.31±238.46 microns (p=0.129), AOD750 reduced to 778.69±262.93 microns (p=0.004), TISA500 reduced to 0.235±0.642 microns (p=0.084), and TISA750 reduced to 0.393±0.117 microns (p=0.025). The mean values of VF MD increased to -12.5±5.85 (p=0.393) and PSD increased to 4.98±4.18 (p=0.083). After surgery, the GCC average reduced to 100.52±13.7 microns (p<0.001), FLV increased to 5.52±6.21 (p=0.088), and GLV increased to 8.48±9.02 (p=0.041) and the ONH RNFL average reduced to 84.35±19.93 microns (p=0.063).

Table 3 shows the correlation between the magnitude of change in ocular parameters and the magnitude of change in average RNFL following SB surgery. It is visible that the magnitude of change in RE (p=0.04), AL (p<0.01), TISA500 (p=0.02), TISA750 (p<0.01), GCC FLV (p=0.02), RNFL IT (p<0.01), TL (p<0.01), TU (p<0.01), and NL (p<0.01) significantly correlated with magnitude of change in RNFL. Thus, after SB surgery, the greater the change in these parameters, the higher is the change in RNFL thickness.

**Table 3:** Correlation between the magnitude of change in ocular parameters with the magnitude of change in average RNFL following SB surgery. *Statistically significant correlation. BCVA: best-corrected visual acuity; AOD: angle opening distance; TISA: trabecular iris surface area; MD: mean deviation; PSD: pattern standard deviation; OCT: optical coherence tomography; GCC: ganglion cell count; FLV: focal loss of volume; GLV: gross loss of volume; ONH: optic nerve head; RNFL: retinal nerve fiber layer; CMT: central macular thickness; RD: retinal detachment; SB: scleral buckling

S. no.	Magnitude of change in parameters post-operative minus (-) pre-operative	Magnitude of change in RNFL post-operative minus (-) pre-operative (non-parametric parameter)	
		r-Value	p-Value
1.	Pre-operative BCVA (log MAR)	-0.01	0.92
2.	Intraocular pressure	0.03	0.84
3.	Cup dimensions	0.13	0.42
4.	Refractive error	-0.31	0.04*
5.	Axial length (mm)	0.42	<0.01*
6.	Anterior chamber depth (mm)	0.26	0.10
7.	AOD 500 (microns)	0.14	0.37
8.	AOD 750 (microns)	0.25	0.11
9.	TISA 500 (microns)	0.35	0.02*
10.	TISA 750 (microns)	0.42	<0.01*
11.	Visual field analysis (dB) MD	-0.15	0.34
12.	Visual field analysis (dB) PSD	-0.08	0.61
13.	OCT GCC average (microns)	0.17	0.29
14.	OCT GCC FLV	0.36	0.02*
15.	OCT GCC GLV	0.06	0.69
16.	OCT ONH RNFL average (microns)	0.04	0.78
17.	OCT RNFL IT (microns)	0.43	<0.01*
18.	OCT RNFL TL (microns)	0.78	<0.01*
19.	OCT RNFL TU (microns)	0.56	<0.01*
20.	OCT RNFL NL (microns)	0.77	<0.01

Table 4 shows an association between qualitative parameters with post-operative thickness of RNFL and with the magnitude of its change following SB surgery. Except for the grade of PVR (p=0.04), none of the qualitative parameters, including extent, type, or location of the buckle, and phakic status had a SS association with post-operative average RNFL thickness or extent of its change. The subject with no PVR had the thickest RNFL (113.8 microns) followed by subjects having PVR B (100.80 microns), PVR C1 (98.85 microns), PVR A (93.60 microns), and lastly, PVR C2 (70.00 microns). Concerning other qualitative parameters, the RNFL was 132.00 microns in 270 degrees, 105.93 microns in 90 degrees, and 101.17 microns in 180-degree buckle; 106.50 microns in 279 type and 103.03 microns in 277 type buckle; 104.50 microns in buckle at 10 mm from the limbus and 103.59 microns in buckle at 8 mm from limbus; and 108.09 microns in phakic, 106.00 microns in aphakic and 97.31 microns in pseudophakic subjects.

**Table 4:** Association between qualitative parameters with post-operative thickness of RNFL and with the magnitude of its change following SB surgery. *SD could not be calculated as only one subject is present in the relevant category. **Statistically significant. RNFL: retinal nerve fiber layer; PVR: proliferative vitreoretinopathy; SB: scleral buckling

			N	Mean RNFL thickness (microns)	SD	p-Value
Extent of buckle (in degrees)	Post-operative RNFL	90	15	105.93	19.352	0.18
		180	24	101.17	16.602	
		270	1	132.00	-*	
	Change in RNFL	90	15	-7.27	19.095	0.15
		180	24	-5.04	14.351	
		270	1	36.00	-*	
Type of buckle 277=1; 279=2	Post-operative RNFL	1	32	103.03	18.964	0.73
		2	8	106.50	14.071	
	Change in RNFL	1	32	-3.91	16.320	0.16
		2	8	-8.63	21.380	
Location of buckle 8/10 mm from limbus	Post-operative RNFL	8	34	103.59	17.129	0.87
		10	6	104.50	24.189	
	Change in RNFL	8	34	-5.97	16.129	0.87
		10	6	1.50	23.365	
Phakic/pseudophakic/aphakic	Post-operative RNFL	Phakic	23	108.09	13.111	0.26
		Pseudophakic	16	97.31	22.700	
		Aphakic	1	106.00	-*	
	Change in RNFL	Phakic	23	-8.04	13.384	0.94
		Pseudophakic	16	-.50	21.765	
		Aphakic	1	-1.00	-*	
PVR	Post-operative RNFL	Nil	16	113.88	11.546	0.04**
		C1	13	98.85	18.366	
		C2	1	70.00	-*	
		A	5	93.60	25.046	
		B	5	100.80	10.060	
	Change in RNFL	Nil	16	-3.75	20.577	0.42
		C1	13	-.38	15.581	
		C2	1	-15.00	-*	
		A	5	-12.80	13.027	
		B	5	-10.00	14.370	

## Discussion

The RD induces photoreceptor degeneration, ganglion cell axon loss, and subsequent retinal ganglion cell (RGC) layer degeneration which may involve even attached retina beyond detachment borders [9,10,17]. The retinal layers suffer due to separation from the choroid, pressure effect of subretinal fluid, and intraocular pressure [18]. The IOP rise and changes the toxic effect of injected gases, retinal dehydration from air infusion, and overall manipulations during surgery damage neural tissue to manifest as RNFL and VF defects [10,19-21]. The final VA is determined by the extent of damage to the macula caused by RD and its corrective surgery. The factors like duration of RD, extent of RD, and post-operative macular changes influence visual functions, and visual loss may be irreversible in macula-off RD despite successful reattachment [5,8].

The thickness of GCC and RNFL is a vital determinant of retinal health [16,22]. Equipment like scanning laser polarimeter [5], Heidelberg retinal tomogram (HRT) [10], and OCT have been utilized to study the RGC layer [9-11]. Compared to conventional OCT, SD-OCT has dramatically improved the visualization of the photoreceptor layer and microstructural changes in the macular area; thus, it can investigate the discordances between anatomic and functional outcomes after RD surgery [23]. The OCT uses low coherence interferometry to produce high-resolution two-dimensional images of optical scattering from internal tissue microstructures [5,10,11]. As it is based on near-infrared interferometry and it is affected less by refractive status, AL, nuclear sclerotic cataract density, and media opacity, OCT has been used to assess the macular microstructural changes following SB in macula-off RRD and has been widely used to measure RNFL thickness in various ocular conditions [23,24]. The SD-OCT measures RNFL thickness from the inner boundary corresponding to the internal limiting membrane to the outer boundary at the level of the RPE [11]. But there are very few studies on RNFL parameters following SB [5,10,11].

In order to find the effect of unilateral primary RRD without PVR in 16 subjects having a mean age of 49.8 years, at 10.3 months after SB, Ozdek et al. compared post-SB RNFL thickness of affected eye with that of the healthy fellow eye using a scanning laser polarimeter (NFA-GDx; San Diego, CA: Laser Diagnostic Technologies) [5]. Among affected eyes, 6/16 (37%) eyes had a history of trauma, 5/16 (31%) eyes had degenerative myopia, and 14/16 (87%) had macular detachment. The SB was done within a mean duration of 28 days of RRD. Following SB, the BCVA decreased in 1/16 eyes, remained unchanged in 2/16 eyes, and increased in 14/16 eyes. As they did not see any macular pathology like cystoid macular edema, macular pucker, or retinal pigment epithelial changes post SB, the RNFL changes may have been the sole responsible factor responsible for the resultant VA. They found that mean post-operative superior average (66.7±15.2 microns), inferior average (74.4±12.4 microns), and average (59.5±7.33 microns) RNFL thickness in operated eyes were less than those of the control eyes (70.8±11.1 microns, p=0.13; 76.9±16.4 microns, p=0.27; and 61.6±10.1 microns, p=0.47, respectively), though the difference was not SS. The authors concluded that SB thinned RNFL through surgical trauma and optic nerve stretching from placement of encircling band. However, there was a significant correlation between RNFL thickness, and with increasing duration of RD (r>0.5, p<0.03). The authors explained that thicker RNFL in operated subjects might have resulted from Muller’s cells proliferation and epiretinal membrane formation and not from the restoration of a healthy status [5].

In a prospective comparative study, Koutsandrea et al. compared post-operative VF loss and RNFL defects in 50 subjects - 25 undergoing SB and 25 undergoing PPV for primary RRD at nine months following successful surgery [10]. The mean age of 50 subjects was about 55 years, and macula was “off” in about 50% of subjects. The mean number of tears was 2.68 for the SB group and 1.88 for the PPV group. The post-operative mean VA and IOP was LogMAR 0.87 ± 0.19 (20/160; 6/48) and 12.4 ± 2.0 mmHg; and LogMAR 0.78 ± 0.22 (20/125; 6/38) and 13±2.3 mmHg in SB and PPV groups, respectively. They examined values of average, supero-temporal, supero-nasal, nasal, inferonasal, inferotemporal, and temporal RNFL. It was observed that the two groups did not significantly differ in RNFL thickness; however, absolute values for RNFL thickness were not given by these authors. The post-operative VF total deviation values were significantly lower (p=0.001) in pre-operatively attached areas of the retina than in detached retina areas and retrospectively were -4.3 ± 3.3 vs -6.9 ± 5.2 in SB and -7.8 ± 5.1 vs. -9.6 ± 5.2 in the PPV group. Though both detachment and trauma during surgery might be affecting different layers of the retina differently, through effect analysis, the authors found that for VF loss, the detachment per se had a much larger role to play than adverse effects of any surgical technique. Neither the absolute values of RNFL thickness were given nor the effect of other ocular parameters on RNFL was found [10]. 

Matlach et al. compared the post-operative thickness of RNFL-GCC-IPL complex in 67 eyes having RRD at 12 months after SB or PPV with 40 healthy controls [11]. Out of 67 eyes, 40 had macula on, and 27 had macula off. The mean age of subjects and the control group was about 63 years. Irrespective of technique, post-surgery, the VA improved significantly (p<0.001) from LogMAR 0.31 ± 0.68 (20/41; 6/12) to LogMAR -0.02 ±0.11 (20/19; 6/4.8) in macula-on group and it was comparable to VA of the control group (LogMAR -0.02±0.07 {20/19; 6/4.8}). In macula-off group, the improvement was less marked and the pre-operative value of LogMAR 1.29 ± 1.11 (20/400; 6/120) improved to LogMAR 0.05 ±0.15 (20/22; 6/7.5) after surgery. The thickest RNFL was found in SN followed by IN quadrant in both surgical groups. Though post-operative average RNFL thickness in operated eyes (125 microns) was found thicker than that in control eyes (120 microns); an incorrectly positioned inner boundary at the epiretinal membrane and vitreomacular tractions caused this. The central macular thickness (CMT) was found in the range of 300-308 microns. These authors found that VA of LogMAR 0.1 (20/25; 6/7.5) or worse correlated with a thicker CMT when the measurement was done with the Cirrus OCT (Pearson’s r =0.57; p = 0.01), but not when RTVue (Pearson’s r=0.11; p=0.67) was used [11].

All the above three studies examined RNFL after a long duration of surgery, i.e., after nine, 10, and 12 months, while RNFL measurement was not done at pre-operative or at immediate post-operative period for comparison with values at three months after SB [5,10,11]. Additionally, the influence of pre-operative and post-operative status of other ocular parameters on RNFL changes was not examined. Ozdek et al. found a significant correlation between duration of RD and RNFL thickness [5], Matlach et al. found that thicker CMT correlated with worse VA [11], and through effect analysis, Koutsandrea et al. concluded that original pathology, i.e., RD itself is responsible for a significant change in RNFL [10].

We found that pre-operative RNFL thickness of 108.58±20.38 microns significantly reduced to 103.73±17.98 microns three months after surgery. In comparison with our study subjects, Ozdek et al. found a very thin RNFL of 59.5±7.33 microns [5], and Matlach et al. found a thicker RNFL of 125 microns [11], while Koutsandrea et al. did not give absolute values of RNFL in their subjects [10]. Despite thinning of RNFL, BCVA improved significantly from LogMAR 2.13±0.44 (20/2000) to LogMAR 1.09±0.4 (20/200; 6/60) in our subjects. The subjects of all other authors had better BCVA as compared to our subjects at the post-operative period [5,10,11]. The probable reason for this may be an earlier intervention with RD surgery (less than seven days [10]; 28 days [5]; 33 days [11]) while the mean duration of RD in our subjects was 19.5±4.7 days before SB. Or it could be due to better pre-operative VA, e.g., it was LogMAR 1.29 ± 1.11 in subjects of Matlach et al. compared to LogMAR 2.13±0.44 in our subjects [11]. Our subjects had a mean IOP of 14.43±3.00 mmHg while subjects of Koutsandrea et al. had lower values of IOP, i.e., 12.4 ± 2.0 mmHg in the SB group [10]. The post-operative CFT in our subjects was 264.55±66.32 microns while it was higher (300-308 microns) in subjects of Matlach et al. who underwent both SB and PPV [11]. This might be due to the difference in values of retinal parameters of different population groups involved [25]. About 50% of our subjects had one tear, 25% had two tears, and the remaining 25% had multiple tears, while the mean number of tears in subjects of Koutsandrea et al. was 2.68 [10]. We had 21 subjects having superior tears and 16 subjects having inferior tears naturally with corresponding buckle placement. We found that RNFL was thickest in IT followed by IN quadrant, and we feel that this thinning of RNFL in superior quadrant was either from pressure effect from buckles or pre se due to tear/hole itself and due to the effect of cryopexy. In context with buckle placement, we made another interesting observation that following SB, the RNFL thickness notably increased in the NL and TL quadrant while reducing in all other quadrants (Table 1). While Ozdek et al. found RNFL to be thickest in inferior average followed by superior average and Matlach et al. found RNFL thickest in SN followed by IN quadrant, but these authors did not give the location of tears/holes, thus the location of buckles cannot be contemplated [5,11]. 

We observed that post-SB average RNFL thickness had a significant correlation with baseline VF MD values, GLV, ONH RNFL, and RNFL thickness of TL, TU, and NL quadrant. The magnitude of change in RNFL had a significant correlation with pre-operative GCC and FLV; and RNFL thickness in IT, TL, TU, and NL quadrants. The ONH RNFL comprises RNFL thickness at 3.45 mm diameter circle centered on the optic nerve head, thus it correlated with overall RNFL thickness, but the latter had higher values than ONH RNFL thickness implying that some fibers lose their vitality during their course to reach the ONH [15]. It is additionally inferred that the temporal quadrant of RNFL may significantly predict the health of overall RNFL. While evaluating RNFL loss following vitrectomy for RD, Takkar et al. also found that percent RNFL change was the highest for temporal quadrant and the change in temporal RNFL was significantly lower in the group with a better visual outcome [26]. In our subjects, the NL quadrant also correlated with average RNFL thickness. We feel it was because buckles were mostly placed in superior (21 subjects) or horizontal (three subjects) quadrants and the lower quadrant thus escaped any pressure effect from explants [6,7]. The GCC comprises the RNFL, the retinal ganglion cell layer, and the inner plexiform layer (thickness of all macular layers between the internal limiting membrane and the inner plexiform layer) [15]; and we found that magnitude of change in RNFL had a significant correlation with pre-operative GCC. Both GLV and FLV had a negative correlation with RNFL parameters implying that higher GCC loss, which may be more marked at the contact point of buckle-band, thinner will be the average RNFL (Table 2). The CFT measured at foveola did not correlate either with post-operative RNFL or with the magnitude of its change, most probably as outer retinal layers are predominant in foveola, and foveola does not have RGC layer (Table 2) [26].

The anterior segment parameters including AL, ACD, AOD, and TISA get altered from SB as the lens-iris diaphragm moves anteriorly, and this may cause a rise in IOP from narrowing of anterior chamber angle. Though a change in these parameters may affect the RNFL thickness, however, this has been rarely studied in the past [27]. In our study, the scleral buckle at 8-10 mm from limbus brought about an irregular change in eye geometry. A myopic shift (p=0.04) and an axial length increase (p<0.01) were accompanied with reduction in AOD500 (p=0.129), AOD750 (p=0.004), TISA500 (p=0.02), and TISA750 (p<0.01), i.e., anterior chamber shallowing. It is clear that elongation is restricted to the posterior segment of the eyeball. We observed that geometry change caused a rise in IOP in a SS manner (Table 1) and the correlation values show that the more the geometry change induced by SB, the more is the thinning of RNFL (Table 3). 

The contact pressure exerted on the globe depends upon the extent (90/180/270 degrees) and type of buckle (277/279), thus the association of these factors was also studied. Additionally, the pressure site varies with buckles placed at 8 mm or 10 mm from the limbus. However, no significant association with these factors was found. It has been found that the human lens has no significant effect on in vivo measurement of RNFL [28]. Thus, RNFL measurements do not differ significantly in phakic or pseudophakic/aphakic subjects. We also found no significant association between phakic/pseudophakic/aphakic status of subjects with RNFL parameters. The PVR is a proliferation of retinal glial cells, retinal pigment epithelium, and inflammatory macrophages into the vitreous through a hole or break, which causes elevation and distortion of the retina. Though PVR is very frequently associated with RD and higher grades of PVR may be detrimental to the health of RNFL, there has not been any study on the association of PVR with RNFL parameters. We found a significant association between grades of PVR and RNFL thickness while observing that with a higher grade of PVR, e.g., C2, the RNFL mean thickness was 70.00 microns, while with no PVR, the RNFL thickness was 113.88 microns.

## Conclusions

In this study, we evaluated RNFL thickness and its changes following SB to study alterations caused by SB and to explain the visual recovery course after an anatomically successful repair of RRD. The predictors of average RNFL thickness following SB include axial length, myopic shift, trabecular iris surface area, visual fields total deviation, and grade of PVR. Out of GCC OCT parameters, average ganglion cell count, focal and gross loss of volume of ganglion cells, and out of RNFL OCT parameters, TU, TL, and NL RNFL affected average RNFL thickness significantly. So, an earlier surgery to prevent ganglion cell and RNFL loss and progression of PVR, optimum length and type of buckle (277/279) to prevent excess pressure effect, and appropriate cryopexy to prevent excessive retinal tissue loss are recommended.

## References

[1] Bock M, Brandt AU, Kuchenbecker J (2012). Impairment of contrast visual acuity as a functional correlate of retinal nerve fibre layer thinning and total macular volume reduction in multiple sclerosis. Br J Ophthalmol.

[2] Parrozzani R, Miglionico G, Leonardi F (2018). Correlation of peripapillary retinal nerve fibre layer thickness with visual acuity in paediatric patients affected by optic pathway glioma. Acta Ophthalmol.

[3] Danesh-Meyer HV, Carroll SC, Foroozan R, Savino PJ, Fan J, Jiang Y, Hoorn SV (2006). Relationship between retinal nerve fiber layer and visual field sensitivity as measured by optical coherence tomography in chiasmal compression. Invest Ophthalmol Vis Sci.

[4] Thompson JT (2013). The effects and action of scleral buckles in the treatment of retinal detachment. Ryan's Retina. Fifth Edition.

[5] Ozdek S, Lonneville Y, Onol M, Gurelik G, Hasanreisoglu B (2003). Assessment of retinal nerve fiber layer thickness with NFA-GDx following successful scleral buckling surgery. Eur J Ophthalmol.

[6] Iwase T, Kobayashi M, Yamamoto K, Yanagida K, Ra E, Terasaki H (2017). Change in choroidal blood flow and choroidal morphology due to segmental scleral buckling in eyes with rhegmatogenous retinal detachment. Sci Rep.

[7] Gama I, Proença H, Gonçalves A (2017). Macular choroidal thickness after vitreoretinal surgery: long-term effect of pars plana vitrectomy with and without encircling scleral buckling surgery. Arch Soc Esp Oftalmol.

[8] La Heij EC, Derhaag PF, Hendrikse F (2000). Results of scleral buckling operations in primary rhegmatogenous retinal detachment. Doc Ophthalmol.

[9] Lee YH, Lee JE, Shin YI, Lee KM, Jo YJ, Kim JY (2012). Longitudinal changes in retinal nerve fiber layer thickness after vitrectomy for rhegmatogenous retinal detachment. Invest Ophthalmol Vis Sci.

[10] Koutsandrea C, Kanakis M, Papaconstantinou D, Brouzas D, Ladas I, Petrou P, Georgalas I (2016). Scleral buckling versus vitrectomy for retinal detachment repair: comparison of visual fields and nerve fiber layer thickness. Ophthalmologica.

[11] Matlach J, Pflüger B, Hain J, Göbel W (2015). Inner and outer central retinal findings after surgery for rhegmatogenous retinal detachment using different spectral-domain optical coherence tomography devices. Graefes Arch Clin Exp Ophthalmol.

[12] Kim M, Park KH, Kim TW, Kim DM (2011). Changes in anterior chamber configuration after cataract surgery as measured by anterior segment optical coherence tomography. Korean J Ophthalmol.

[13] Radhakrishnan S, Goldsmith J, Huang D (2005). Comparison of optical coherence tomography and ultrasound biomicroscopy for detection of narrow anterior chamber angles. Arch Ophthalmol.

[14] Odrobina D, Laudańska-Olszewska I, Gozdek P, Maroszyński M, Amon M (2013). Influence of scleral buckling surgery with encircling band on subfoveal choroidal thickness in long-term observations. Biomed Res Int.

[15] Garas A, Vargha P, Holló G (2010). Reproducibility of retinal nerve fiber layer and macular thickness measurement with the RTVue-100 optical coherence tomograph. Ophthalmology.

[16] Arvanitaki V, Tsilimbaris MK, Pallikaris A, Moschandreas I, Minos E, Pallikaris IG, Detorakis ET (2012). Macular retinal and nerve fiber layer thickness in early glaucoma: clinical correlations. Middle East Afr J Ophthalmol.

[17] de Souza CF, Kalloniatis M, Polkinghorne PJ, McGhee CN, Acosta ML (2012). Functional and anatomical remodeling in human retinal detachment. Exp Eye Res.

[18] Işik Ü, Kaygisiz M (2020). Assessment of intraocular pressure, macular thickness, retinal nerve fiber layer, and ganglion cell layer thicknesses: ocular parameters and optical coherence tomography findings in attention-deficit/hyperactivity disorder. Braz J Psychiatry.

[19] Uemura A, Kanda S, Sakamoto Y, Kita H (2003). Visual field defects after uneventful vitrectomy for epiretinal membrane with indocyanine green-assisted internal limiting membrane peeling. Am J Ophthalmol.

[20] Doi M, Ning M, Semba R, Uji Y, Refojo MF (2000). Histopathologic abnormalities in rabbit retina after intravitreous injection of expansive gases and air. Retina.

[21] Kokame GT (2000). Visual field defects after vitrectomy with fluid-air exchange. Am J Ophthalmol.

[22] Ocansey S, Abu EK, Owusu-Ansah A (2020). Normative values of retinal nerve fibre layer thickness and optic nerve head parameters and their association with visual function in an African population. J Ophthalmol.

[23] Huang C, Fu T, Zhang T, Wu X, Ji Q, Tan R (2013). Scleral buckling versus vitrectomy for macula-off rhegmatogenous retinal detachment as accessed with spectral-domain optical coherence tomography: a retrospective observational case series. BMC Ophthalmol.

[24] Schuman JS, Pedut-Kloizman T, Hertzmark E (1996). Reproducibility of nerve fibre layer thickness measurements using optical coherence tomography. Ophthalmology.

[25] Pradhan ZS, Braganza A, Abraham LM (2013). Determinants of macular thickness in normal Indian eyes. J Clin Ophthalmol Res.

[26] Takkar B, Azad R, Kamble N, Azad S (2018). Retinal nerve fiber layer changes following primary retinal detachment repair with silicone oil tamponade and subsequent oil removal. J Ophthalmic Vis Res.

[27] Khanduja S, Bansal N, Arora V, Sobti A, Garg S, Dada T (2015). Evaluation of the effect of scleral buckling on the anterior chamber angle using ASOCT. J Glaucoma.

[28] Collur S, Carroll AM, Cameron BD (2000). Human lens effect on in vivo scanning laser polarimetric measurements of retinal nerve fiber layer thickness. Ophthalmic Surg Lasers.

